# Driving Force of Covid-19 Among People Living With HIV/AIDS in Wuhan, China

**DOI:** 10.21203/rs.3.rs-53351/v1

**Published:** 2020-08-10

**Authors:** Wei Guo, Fangzhao Ming, Yu Dong, Qian Zhang, Lian Liu, Ming Gao, Xiaoxia Zhang, Pingzheng Mo, Yong Feng, Weiming Tang, Ke Liang

**Affiliations:** Department of Pathology, Zhongnan Hospital of Wuhan University, Wuhan, 430071, Hubei, China; Wuchang District Center for Disease Control and Prevention, Wuhan, 430060, Hubei, China; Department of Geriatrics, Zhongnan Hospital of Wuhan University, Wuhan, 430071, Hubei, China; Qingshan District Center for Disease Control and Prevention, Wuhan, 430080, Hubei, China; Caidian District Center for Disease Control and Prevention, Wuhan, China; Xinzhou District Center for Disease Control and Prevention, Wuhan, China; Department of Infectious Diseases, Zhongnan Hospital of Wuhan University, Wuhan, 430071, Hubei, China; Department of Infectious Diseases, Zhongnan Hospital of Wuhan University, Wuhan, 430071, Hubei, China; Department of Medical Microbiology, School of Basic Medical Sciences, Wuhan University, Wuhan, 430071, Hubei, China; Dermatology Hospital, Southern Medical University, Guangzhou, China; Department of Infectious Diseases, Zhongnan Hospital of Wuhan University, Wuhan, 430071, Hubei, China

**Keywords:** COVID-19, People living with HIV/AIDS (PLWHA), Morbidity, Risk Factor

## Abstract

**Background:**

Even people living with HIV/AIDS (PLWHA) were considered to be at increased risk of SARS-CoV-2 infection, the driving force among this group of individuals is still not clear.

**Methods:**

We investigated 1,701 PLWHA through a telephone interview and found 11 COVID-19 patients in four districts of Wuhan, China. The demographic features and major clinical characteristics of these patients were retrieved from the information management systems for COVID-19 patients of four districts’ CDC. Statistical analysis was performed to find out the driving force of COVID-19 among PLWHA.

**Results:**

The incidence proportion of COVID-19 in PLWHA is 0.6% (95% CI: 0.2% - 1.0%), which is comparable to the overall population incidence rate in Wuhan city (0.6%). Nine out of the 11 COVID-19/AIDS patients had relatively high CD4+ T lymphocyte count (>200/μl) and undetectable HIV viral load (20 copies/ml), and ten of them were on antiretroviral therapy. PLWHA who were old, had low CD4+ T lymphocyte count, infected HIV through homosexual activity, and had been diagnosed for HIV for a long time, were more likely to develop COVID-19.

**Conclusions:**

PLWHA has comparable COVID-19 morbidity rates as the general population, and older age, low CD4 count, long length since HIV diagnosis, and treatment-naive were potential driving forces of COVID-19 occurrence among PLWHA. Strategies in preventing SARS-CoV-2 infection among PLWHA with worse immune responses are needed.

**Article Summary Line:**

As COVID-19 continues to spread around the world, people living with HIV/AIDS (PLWHA) are also at risk of infection with SARS-CoV-2. We investigated the factors associated with SARS-CoV-2 infection among PLWHA in Wuhan, China.

## Background

As a high contagious pathogen, Severe Acute Respiratory Syndrome Coronavirus 2 (SARS-CoV-2) rapidly spread around the world, and lead to the death of 690,953 by August 4th. In response to the emerging infectious diseases, a large number of studies had been conducted to summarize the clinical characteristics of COVID-19. Those studies have summarized that chronic diseases, such as hypertension, chronic pulmonary diseases, and diabetes, etc., are the driving force of both morbidity and fatality of COVID-19[1, 2]. However, up till now, to the best of our knowledge, very few studies have been conducted to evaluate the driving forces of SARS-CoV-2 infection among people living with HIV/AIDS (PLWHA), while previous studies indicated that PLWHA were presumed to be at a higher risk of SARS-CoV-2 infection as their compromised immunity. Further investigation of the driving force of SARS-CoV-2 infection among PLWHA may help us to better protect this vulnerable group.

Since the first confirmed case was reported in Wuhan, as the first epidemic center of the pandemic, Wuhan provided a unique opportunity to further investigate the driving forces of COVID-19 among PLWHA. Between December 31st of 2019 and May 14th of 2020, an accumulative of 84,464 confirmed cases were reported in China, while 50,339 of them were reported in Wuhan, 3,869 died. Thus, we summarized the situation of PLWHA in four districts of Wuhan and reached all the PLWHA who are on care in the four districts.

In this study, we investigated the incidence proportion of COVID-19 among PLWHA and evaluated the potential factors associated with the development of COVID-19 among PLWHA.

## Materials And Methods

### Patients in four districts of Wuhan

**Figure 1:**
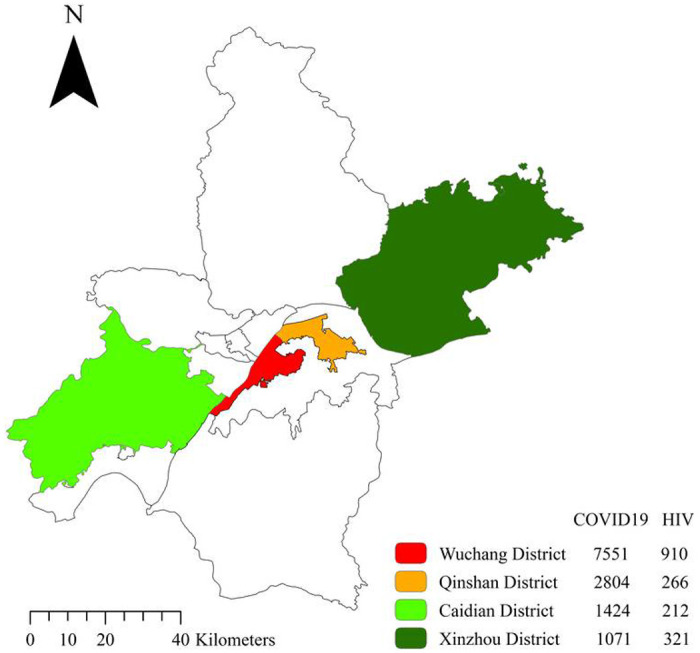
The distribution of patients with COVID-19 and patients with HIV/AIDS in four districts, Wuhan. The distribution of all documented, laboratory-confirmed cases of coronavirus disease 2019 (COVID-19), and all documented patients with HIV/AIDS in Wuchang, Qingshan, Caidian and Xinzhou n districts, Wuhan was shown in the figure, according to the official management system of Center for Disease Control and Prevention of Hubei province by May 14,2020. Note: The designations employed and the presentation of the material on this map do not imply the expression of any opinion whatsoever on the part of Research Square concerning the legal status of any country, territory, city or area or of its authorities, or concerning the delimitation of its frontiers or boundaries. This map has been provided by the authors.

Up till 14th May, the endpoint of the follow-up, the total number of COVID-19 patients in Wuchang, Qingshan, Caidian and Xinzhou districts was 7,551 (15.0% in Wuhan), 2,804 (5.6% in Wuhan), and 1,424 (2.8% in Wuhan) and 1,071(2.1% in Wuhan), respectively[3]. There were 5,953 PLWHA on care in Wuhan, while 1,709 PLWHA were managed by the four districts Center for Disease Control and Prevention (CDC), including 910 (15.3% in Wuhan) in Wuchang, 266 (4.5% in Wuhan) in Qingshan, 321 (5.4% in Wuhan) in Xinzhou, and 212 (3.7% in Wuhan) in Caidian, respectively (Fig. 1).

### The scheme of the investigation

PLWHA were investigated through a telephone call or social communication software investigation (16th Feb-14th May) because of the lockdown of the whole city (23rd Jan-18th Apr). Whether the patients had typical symptoms mentioned in the previous clinical reports, such as fever, non-productive cough, dyspnea, etc.[4], were inquired. The contact history with confirmed or suspected COVID-19 patients was then investigated. For those who presented with clinical symptoms or contacted COVID-19 patients, they were introduced to a local designated hospital for CT scan and nucleic acid test (NAT) for SARS-CoV-2. Diagnosis criteria for COVID-19 were according to the Diagnosis and Treatment of COVID-19 in China (the 7th edition) [5].

All the questionnaires were confirmed by face to face investigation from April 18th to May 14th. We also double-checked the name and identification card number of each enrolled PLWHA and that of the COVID-19 patient in the information management systems of four districts’ Center for Disease Control and Prevention (CDC). The clinical records were retrieved from the corresponding hospitals, and the features of COVID-19 cases in PLWHA had been reported[6].

### Laboratory results acquisition

The NATs for SARS-COV-2 were performed in the local designated hospital. The usage of laryngeal swab specimens and the real-time reverse-transcription polymerase chain reaction were reported previously[4]. The CT and NAT results were retrieved from the information management systems for COVID-19 patients of four districts’ CDC. The patients’ most recent (in 3 months) CD4 + T lymphocyte count (CD4 count) and HIV viral load (HIV-VL), the current regimen of ART, and other demographics information were obtained from the AIDS Comprehensive Prevention and Control Data Information Management System of the Chinese CDC.

### Statistical analysis

Categorical variables were presented as count (%), and continuous variables were presented as mean ± standard deviation. Univariate and multivariable logistic regressions were used to identify the factors associated with COVID-19 among PLWHA, while age, gender, marriage status, transmission route of HIV, CD4 count, HIV-VL, number of days since diagnosed, number of days since on ART, and ART regimen were included in the multivariable logistic regression model. Odds ratios (OR) and adjusted odds ratio (AOR) with 95% confidence intervals (CI) and P-values were reported. As both CD4 count and HIV-VL were not normally distributed, they were further undergone log transformation. We performed all statistical analyses using SAS 9.4.

#### Ethical approval

This study was approved by the Zhongnan Hospital of Wuhan University institutional review board (2020079K), and the informed consent was waived.

## Results

### Patients enrolled in the study

Overall, a total of 1,709 PLWHA in the four study districts were reached and investigated. Of whom, five were living outside of Wuhan for more than six months, and three were asymptomatic carriers (NAT positive and CT negative), and thus were ruled out from the study (Figure 1). The average age of the included 1,701 participants was 42±14.5 years old, 1484 (87.2%) were male, and 217 (12.8%) were female. As for ART, most of PLWHA (1,406, 82.7%) took Nucleoside Reverse Transcriptase Inhibitors (NRTIs) and Non-Nucleoside Reverse Transcriptase Inhibitors (NNRTIs) as the regimen, while 172 (10.1%) took LPV/r-based ART, 87(5.1%) took integrase inhibitors(INI) based ART (62 Dolutegravir-based, 19 Elvitegravir/Cobicista-based, 4 Raltegravir-based, 2 Bictegravir-based). To be noted, there were still 36 individuals (2.1%) who were still treatment naive.

### The morbidity for COVID-19 in PLWHA

By screening investigation and further retrieval of the medical histories from CDC, we found 11 confirmed COVID-19 patients among the 1,701 participants. The morbidity of COVID-19 in PLWHA was about 0.6% (95%CI: 0.2%-1.0%). Among the 11 COVID-19 patients, ten (90.9%) males with HIV infection through homosexual transmission route, and one (9.1%) was female who got HIV via heterosexual transmission route. The average timing length from the HIV diagnosis was 2,740±1,140 days, and the average length from the ART initiative was 2,030±1,040 days, respectively. Nine out of the 11 COVID-19/AIDS patients had relatively high CD4 count (>200/μl) and undetectable HIV viral load (20 copies/ml). There were 81.8% (nine cases) COVID-19/AIDS took NRTI+NNRTIs, one took LPV/r-based ART, one did not take any ART. There was no significant difference in the morbidity of COVID-19 between the patients taking different ART regimens (Table 1).

According to the Diagnosis and Treatment of COVID-19 in China (the 7^th^ edition)[5], six of the COVID-19 patients were categorized as mild cases, three as severe cases, and two were critical cases, who were died later on.

### Factors associate with COVID-19 among PLWHA

As shown in Table 1, we compared the characteristics of PLWHA with COVID-19 or not. The average age of these COVID-19 patients (n=11) was 53.2±14.5 years old, which was significantly (P=0.012) older than the age of those without COVID-19 (n=1,690). Both univariate and multivariable logistic regression analyses indicated that old age was associate with the occurrence of COVID-19 (AOR=1.07, 95% CI:1.02, 1.13) (Table 2).

The multivariable results also indicated that the log transferred CD4 count was negatively associated with the occurrence of COVID-19 (AOR=0.06, 95%: CI 0.01-0.30), while log transferred length from HIV diagnosis was positively associated with the occurrence of COVID-19 (AOR=1.12, 95% CI:1.05-1.19). Besides, we also found that people who were infected HIV through heterosexual transmission route was less likely to develop COVID-19, as compared to homosexual transmission (AOR=0.07, 95% CI: 0.01-0.90). Results from the multivariable logistic regression analysis also suggested treatment naïve was also marginally associated with COVID-19 occurrence, as compared to treatment with NRTI+NNRTIs regimen (AOR=13.36 95% CI: 0.77-231.74) (Table 2).

## Discussion

Immunocompromised individuals are assumed more susceptible to SARS-CoV-2 infection. While PLWHA presented different extent immune suppress characteristics, this study may help us to identify evidence in how to prevent COVID-19 not only among PLWHA but also among other immune-compromised individuals. Our study extended the existing literature by assessing the incidence proportion of COVID-19 among PLWHA and exploring the driving forces of COVID-19 occurrence among PLWHA. We found that age, CD4 count, time length since HIV diagnosis and treatment status were associated with COVID-19 occurrence among PLWHA.

In the current investigation, we found that the PLWHA exhibits comparable COVID-19 morbidity with the general population in Wuhan (~0.6%, 50 thousand/9 million by 14^th^ May. 2020). This relatively low morbidity in PLWHA might partially attribute to high treatment coverage, as 98% of them were already on ART, and treatment naive was one potential driving force of the occurrence of COVID-19. Effective ART improves the immune system, and most of the CD4 count of those with regular ART was at a relatively normal count (over 200/μL), which might reduce the chance of opportunistic infection[7]. The strict quarantine at the epidemic center of Wuhan may also contribute to this low and comparable disease morbidity rate[8,9]. In order to avoid exposure to SASA-CoV-2, multiple strategies were implemented in Wuhan for PLWHA. For example, to prevent PLWHA run out of antiviral drugs and to keep them away from the high contagious infectious hospitals that admitted a large number of COVID-19 cases, one community-based organization in Wuhan arranged volunteers to collect drugs on behalf and deliver the drugs to PLWHA individually[10].

We found that homosexual transmission is considered to be a risk factor of succumbing to COVID-19. Consistently, in the previous case series, all the COVID-19/AIDS cases appeared homosexually transmitted[11]. The reasons for this phenomenon are unclear yet, further investigations about the behavioristics of patients transmitted by both homosexual and heterosexual were in need.

We found that people with lower CD4 count were more likely to develop COVID-19, although there were no studies on driving forces in HIV negative population, CD4 count decrease, and lymphopenia were reported to correlate with the severity of COVID-19[12–14]. Recent researches indicated that lymphocytes, and especially CD4+ T lymphocytes, are pivotal in the dynamic anti-SARS-CoV-2 immune responses[15,16], i.e., early CD4 responses were considered to be protective against SARS-CoV-2, while late phase CD4 responses amplified pathological inflammation. Moreover, our findings indicated that the long length from HIV diagnosis is also correlated with the occurrence of COVID-19, which may speculate that the long term exhausting of lymphocytes in PLWHA account for an imbalanced immune state and low CD4 count, even after regular ART, which makes the individuals more vulnerable to SARS-CoV-2 infection[17, 18]. Further studies are required to elucidate the immune-pathogenesis in COVID-19/PLWHA.

In this study, the age of COVID-19/PLWHA was older than the other PLWHA, which was consistent with that in the overall COVID-19 population, i.e., the older males had a higher proportion in the COVID-19 patients [2]. Furthermore, our study suggested that older age was a risk factor for HIV/AIDS suffered COVID-19, which was also similar to the HIV negative population[19].

Our study has several limitations. First, even our study included 1701 individuals, only 11 COVID-19 cases were included, which may limit our power to detect the driving force of COVID-19 among PLWHA, and our results need to be explained by caution. Second, the transmission chains of the COVID-19 cases could not be textual researched, which impede our further speculating the reasons for homosexual transmitted patients presented with a higher risk of COVID-19. Last but not least, although we found three asymptomatic carriers by chance, there should be more unrevealed virus carriers due to the speculated higher infection rate in PLWHA. To evaluate the proportion of COVID-19 patients in all SARS-CoV-2 carriers will help us to reveal the immune-pathogenesis of COVID-19.

## Conclusion

PLWHA has comparable COVID-19 morbidity rates as the general population, and older age, low CD4 count, long length since HIV diagnosis, and treatment-naive were potential driving forces of COVID-19 occurrence among PLWHA. Strategies in preventing SARS-CoV-2 infection among PLWHA with worse immune responses are needed.

## Figures and Tables

**Figure 2:**
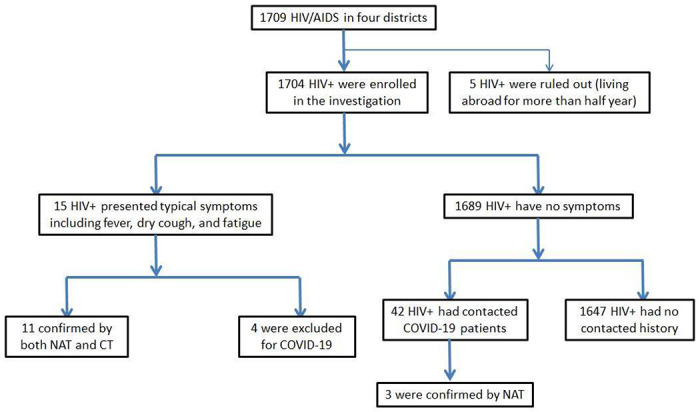
The scheme of the investigation. The investigation followed the scheme shown here. HIV/AIDS patients enrolled in this cohort are documented in the AIDS Comprehensive Prevention and Control Data Information Management System of the Chinese Center for Disease Control and Prevention.

**Table 1.** Social-demographic and status of people living with HIV in Wuhan, China, 2020 (N=1701)

	Total enrolled HIV/AIDS (n=1701)	COVID-19 (n=11)	Without COVID-19 (n=1690)
Age	42.0±14.5	53.2±12.8	42.0±14.5
Gender			
*Male*	1484(87.2%)	10 (90.9%)	1474(87.2%)
*Female*	217 (12.8%)	1 (9.1%)	216 (12.8%)
Marriage status			
*Married*	416 (24.5%)	4 (36.4%)	412 (24.4%)
*Unmarried*	854 (50.2%)	4 (36.4%)	850 (50.3%)
*Widow*	431 (25.3%)	3 (27.3%)	428 (25.3%)
Transmission route			
*Homosexual*	1173 (69.0%)	10 (90.9%)	1163 (68.8%)
*Heterosexual*	495 (29.1%)	1 (9.1%)	494 (29.2%)
*Others*	33 (1.9%)	0 (0.0%)	33 (2.0%)
Log10(CD4)*	2.63±0.31	2.50±0.42	2.63±0.31
Log10(VL)*	0.89±1.62	0.41±1.37	0.89±1.62
Days diagnosed/100	16.8±11.8	27.4±11.4	16.8±11.7
Days ART/100	14.5±10.4	20.3±10.4	14.5±10.4
ART regimen			
*NRTI+NNRTI*	1406(82.7%)	9 (81.8%)	1397(82.7%)
*LPV/r-based*	172 (10.1%)	1 (9.1%)	171 (10.1%)
*INI-based*	87 (5.1%)	0 (0.0%)	87 (5.1%)
*None*	36 (2.1%)	1 (9.1%)	35 (2.1%)

Note:

* Most recent, INI: integrase inhibitors; INI-based: 62 Dolutegravir-based, 19 Elvitegravir/Cobicista-based, 4 Raltegravir-based, 2 Bictegravir-based.

**Table 2.** Factors associate with COVID-19 occurrence among people living with HIV in Wuhan, China, 2020 (N=1701)

	Univariable Firth’s Logistic Regression OR (95% CI)	P	Multivariable Firth’s Logistic Regression AOR (95% CI)	P
Age	1.05 (1.01, 1.09)	0.012	1.07 (1.02, 1.13)	0.010
Gender				
Male	1		1	
Female	0.97 (0.17, 5.44)	0.975	3.00 (0.17, 52.01)	0.451
Marriage status				
*Married*	1		1	
*Unmarried*	0.49 (0.13, 1.80)	0.280	0.96 (0.17, 5.23)	0.958
Widow	0.75 (0.18, 3.05)	0.687	0.51 (0.14, 1.87)	0.312
Transmission route				
*Homosexual*	1		1	
*Heterosexual*	0.34 (0.06, 1.87)	0.213	0.07 (0.01, 0.90)	0.042
*Others*	1.65 (0.09, 30.02)	0.734	0.003 (<0.001, 0.85)	0.044
Log10(CD4)*	0.34 (0.12, 0.97)	0.044	0.06 (0.01, 0.30)	<0.001
Log10(VL)*	0.86 (0.55, 1.33)	0.486	0.62 (0.33, 1.16)	0.133
Days diagnosed/100	1.06 (1.02, 1.10)	0.002	1.12 (1.05, 1.19)	<0.001
Days ART/100	1.05 (1.00, 1.09)	0.040	0.97 (0.90, 1.04)	0.318
ART regimen				
*NRTI+NNRTI*	1		1	
*LPV/r-based*	1.29 (0.23, 7.28)	0.776	1.25 (0.25, 6.29)	0.786
*INI-based*	0.84 (0.05, 14.79)	0.906	1.84 (0.12, 29.37)	0.666
*None*	6.22 (1.05, 36.68)	0.044	13.36 (0.77, 231.74)	0.075

Note: OR=odds ratio, AOR=adjusted odds ratio, CI=confidence interval;

* Most recent testing.
